# Experimentally Verified Parameter Sets for Modelling Heterogeneous Neocortical Pyramidal-Cell Populations

**DOI:** 10.1371/journal.pcbi.1004165

**Published:** 2015-08-20

**Authors:** Paul M. Harrison, Laurent Badel, Mark J. Wall, Magnus J. E. Richardson

**Affiliations:** 1 MOAC Doctoral Training Centre, University of Warwick, Coventry, United Kingdom; 2 School of Life Sciences, University of Warwick, Coventry, United Kingdom; 3 Warwick Systems Biology Centre, University of Warwick, Coventry, United Kingdom; 4 Laboratory for Circuit Mechanisms of Sensory Perception, RIKEN Brain Science Institute, Wako, Saitama, Japan; Indiana University, UNITED STATES

## Abstract

Models of neocortical networks are increasingly including the diversity of excitatory and inhibitory neuronal classes. Significant variability in cellular properties are also seen within a nominal neuronal class and this heterogeneity can be expected to influence the population response and information processing in networks. Recent studies have examined the population and network effects of variability in a particular neuronal parameter with some plausibly chosen distribution. However, the empirical variability and covariance seen across multiple parameters are rarely included, partly due to the lack of data on parameter correlations in forms convenient for model construction. To addess this we quantify the heterogeneity within and between the neocortical pyramidal-cell classes in layers 2/3, 4, and the slender-tufted and thick-tufted pyramidal cells of layer 5 using a combination of intracellular recordings, single-neuron modelling and statistical analyses. From the response to both square-pulse and naturalistic fluctuating stimuli, we examined the class-dependent variance and covariance of electrophysiological parameters and identify the role of the h current in generating parameter correlations. A byproduct of the dynamic *I-V* method we employed is the straightforward extraction of reduced neuron models from experiment. Empirically these models took the refractory exponential integrate-and-fire form and provide an accurate fit to the perisomatic voltage responses of the diverse pyramidal-cell populations when the class-dependent statistics of the model parameters were respected. By quantifying the parameter statistics we obtained an algorithm which generates populations of model neurons, for each of the four pyramidal-cell classes, that adhere to experimentally observed marginal distributions and parameter correlations. As well as providing this tool, which we hope will be of use for exploring the effects of heterogeneity in neocortical networks, we also provide the code for the dynamic *I-V* method and make the full electrophysiological data set available.

## Author Summary

Neurons are the fundamental components of the nervous system and a quantitative description of their properties is a prerequisite to understanding the complex structures they comprise, from microcircuits to networks. Mathematical modelling provides an essential tool to this end and there has been intense effort directed at analysing networks constructed from different classes of neurons. However, even neurons from the same class show a broad variability in parameter values and the distributions and correlations between these parameters are likely to significantly affect network properties. To quantify this variability, we used a combination of intracellular recording, single-neuron modelling, and statistical analysis to measure the physiological variability in pyramidal-cell populations of the neocortex. We employ protocols that measure parameters from both square-pulse and naturalistic stimuli, characterising the perisomatic integration properties of these cells and allowing for the straightforward extraction of mathematically tractable reduced neuron models. We provide algorithms to generate populations of these neuron models that respect the parameter variability and co-variability observed in our experiments. These represent novel tools for exploring heterogeneity in neocortical networks that will be useful for subsequent theoretical and numerical studies. Finally, we make our full electrophysiological dataset available for other research groups to extend and improve on our analysis.

## Introduction

The neocortex is a laminar structure comprising a diversity of neuronal types, of which the principal neurons are pyramidal cells (for a review see reference [1]). Understanding how the properties of the distinct neuronal classes interact to support and govern neocortical computational processes is a key challenge in neuroscience. Combined with acquisition of massive data sets, computational modelling and simulation are now central tools in the study of how the components of neural tissue interact across the genetic, cellular and functional levels [2–5].

In parallel with computer simulations, the mathematical framework required for a more general understanding of how cellular properties such as synaptic filtering [6, 7], synaptic dynamics [8, 9], voltage-gated or calcium-gated currents [10–12] and spike-frequency adaptation [13, 14] can affect network dynamics has also been extensively developed. Though these methods account for a diversity of biophysical detail, the focus has often been on networks featuring one or a few homogeneous populations comprised of neurons with the same level of expression of a particular biophysical characteristic.

Nominally uniform populations, like those of neocortical pyramidal cells, feature a significant variability in their electrophysiology [15–20] and there is increasing theoretical interest in the effects of heterogeneity on populations and networks of both excitatory and inhibitory neurons [21–27]. The typical responses of heterogeneous networks are not necessarily the same as those of homogeneous networks comprised of typical neurons and studies have shown significant differences in synchronization properties [28, 29], coding efficiencies [30, 31] and gain [27] in the presence of heterogeneity.

Though there exist many experimental studies on heterogeneity, the data are not always in a form that allows for the construction of neuron models with the correct parameter distributions and correlations. As a result theoretical studies have tended to focus on a particular parameter (e.g. input resistance) and give it some plausible distribution across the neuronal population. However, such heuristics might not always reflect the empirical distributions well for a particular variable and are rarely combined with heterogeneity in other potentially covarying variables. To address the availability of experimental data that are in suitable form for modelling heterogeneities of pyramidal-cell integration, we measured a range of perisomatic electrophysiological properties of somatosensory cortex layer-2/3, layer-4, and slender-tufted and thick-tufted layer-5 pyramidal cells using standard and dynamic *I-V* [32] stimulation protocols delivered during whole-cell somatic patch-clamp recording. A useful byproduct of this approach is the direct generation of reduced neuron models that accurately reproduce empirical voltage timecourses and can be employed in the mathematical analysis of networks.

For all classes of pyramidal cell examined the current integration at the soma, as encoded by the dynamic *I-V* curve, was found to take the exponential integrate-and-fire (EIF) form, but with class-dependent parameter statistics. We present a systematic analysis of the measured parameter variance and covariance for the different classes of pyramidal cell studied and investigate the major sources of variability in the dataset using principal component analysis. We then provide algorithms for generating the distinct EIF-model parameter sets that respect the correct marginal distributions and correlation structure of our dataset. Networks comprised of these model neurons may be numerically simulated and are also amenable to theoretical study. Our algorithms therefore represent a novel tool for the analysis of heterogeneous networks with experimentally verified parameter distributions.

As part of the Supporting Information we also provide computer code for the experimental dynamic *I-V* curve method together with example data sets. To facilitate other research groups improving on our modelling approach we also published the full experimental data set at www.datadryad.org (doi:10.5061/dryad.d30k7).

## Materials and Methods

### Ethics statement

All experiments were performed in accordance with the UK Animals (Scientific Procedures) Act (1986).

### Preparation of neocortical slices

Parasagittal slices of somatosensory neocortex (300 *μ*m) were prepared from male Wistar rats, at postnatal day 16–18. Rats were kept on a 12 hour light-dark cycle with slices made 90 minutes after entering the light cycle. In accordance with the UK Animals (Scientific Procedures) Act (1986), rats were killed by cervical dislocation and then decapitated. The brain was rapidly removed, cut down the midline and the two sides of the brain stuck down. The brain was angled at 15° so that planar slices could be obtained with the dendritic structure of the excitatory neurons intact. Slices were cut with a Microm HM 650 V micro-slicer (Carl Zeiss) in cold (2–4°C) high Mg^2+^ low Ca^2+^ artificial cerebrospinal fluid (aCSF) consisting of 127 mM NaCl, 1.18 mM KH_2_PO_4_, 2.14 mM KCl, 26 mM NaHCO_3_, 8 mM MgCl_2_, 0.5 mM CaCl_2_ and 10 mM glucose. Slices were stored at 34°C for 1 hour in standard aCSF (1 mM Mg^2+^and 2 mM CaCl_2_) and then at room temperature for 1–6 hours.

### Patch-clamp recordings from excitatory cells

**Fig 1:**
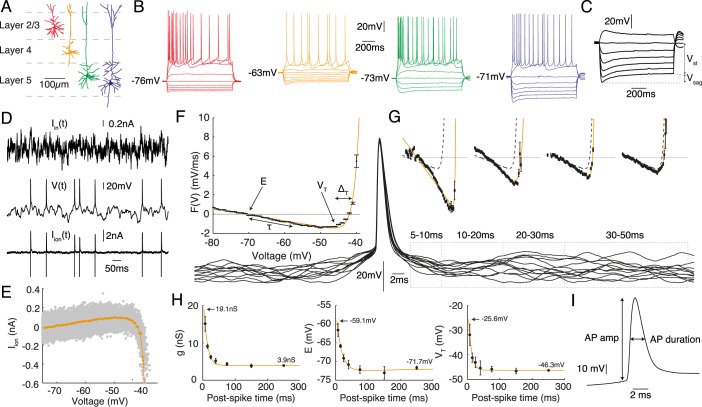
The four cell classes studied were **A** layer-2/3 (red), layer-4 (orange), slender-tufted layer-5 (green) and thick-tufted layer-5 (blue) pyramidal neurons. **B** Representitive intracellular-voltage response to 1 s step currents. **C** Sag ratio *V*_sag_/*V*_st_ (expressed as a percentage). **D-I** Parameters measured from the response to fluctuating-current stimulation using the dynamic *I* − *V* curve protocol. **D** A layer-4 pyramidal cell stimulated by fluctuating current *I*_in_(*t*) with the voltage *V*(*t*) recorded. The ionic current *I*_ion_(*t*) estimated from the difference between stimulation and capacitance currents. **E** Scatter plot of *I*_ion_ versus voltage (grey) and dynamic-*I-V* curve *I*_dyn_ (orange) with data 200 ms following each spike excluded. **F** Steady-state forcing function *F*(*V*) = −*I*_dyn_/*C* (black points) with fit (orange) to the exponential integrate-and-fire (EIF) model Eq (2). The roles of the various parameters are illustrated. **G** Empirical forcing terms (black points) measured in post-spike time slices (spike-triggered voltage traces are shown below) with fits to the EIF model (orange). The steady-state *F*(*V*) from panel F is also plotted (dashed lines) for comparison. **H** Parameter dynamics from the post-spike *F*(*V*) fits for conductance *g* (mono-exponential fit, orange), resting potential *E* (bi-exponential fit due to weak sag,) and spike-onset threshold *V*_T_ (mono-exponential fit, orange). Errorbars from bootstrap resampling. **I** Mean action-potential shape parameters extracted from isolated spikes.

A slice was transferred to the recording chamber and perfused at 2 ml/min with aCSF at 32°C. Slices were visualised using an Olympus BX51W1 microscope with IR-DIC optics and a Hitachi CCD camera (Scientifica, Bedford, UK). Whole-cell recordings were made with patch pipettes (5–8 mΩ) manufactured from thick walled glass (Harvard Apparatus Edenbridge UK) containing 135 mM K-gluconate, 7 mM NaCl, 10 mM HEPES, 0.5 mM EGTA, 2 mM ATP, 0.3 mM GTP, and 10 mM phosphocreatine (290 mOSM, pH 7.2). Voltage recordings were obtained using an Axon Multiclamp 700B amplifier and digitised at 20 kHz with a Digidata 1440A (Molecular Devices, Sunnyvale, CA). Pyramidal neurons were identified based on their location in the layered neocortex, somata size and dendritic extent (Fig 1A). The liquid-junction potential was not compensated for. During recording, neurons were labelled either with the fluorescent dye Alexa Fluor® 488 hydrazide (12.5 mM, Life Technologies, Paisley, UK) or with biocytin (1 mg/mL, Sigma-Aldrich, Dorset, UK) to allow confirmation of the cell type and to ensure an intact apical dendrite.

### Stimulation protocols

Cells were stimulated with square-pulse currents (Fig 1B-1C) and naturalistic *in vivo*-like currents (Fig 1D), during which the typical access resistance was 9–13 MΩ. The form of the naturalistic currents injected consisted of two summed Ornstein-Uhlenbeck processes with time constants *τ*_fast_ = 3 ms and *τ*_slow_ = 10 ms representing AMPA and GABA_A_ time-courses respectively [32]. Two sets of variances, a low (*σ*_slow_ = 0.18 = *σ*_fast_) and a high (*σ*_slow_ = 0.25, *σ*_fast_ = 0.36), along with two DC biases (0.5 and 1) were used giving four distinct current traces for each recording. Finally, a multiplicative gain factor (in the range 200 pA–2000 pA) was applied to the whole of each current so that the firing rate was between 5–15 Hz. Due to high-frequency components in the stimuli, standard techniques compensating for electrode filtering were insufficient and therefore the resulting artefacts were removed using post-recording defiltering techniques [32, 33].

### Histology and confocal microscopy

On removal from the bath the slice was immediately transferred to a 4% paraformaldehyde solution (PBS, pH 7.3) and incubated overnight at ∼ 4°C. Images of fluorescently labelled neurons were obtained using a Leica SP5 confocal microscope. Standard processing was used for biocytin labelled neurons and cells were visualised with the DAB reaction mixture. The reaction was quenched when the dendritic structure of the cells became visible under visual inspection using tris buffer solution (TBS, pH 7.3).

### *I*_h_-channel blocking experiments

To investigate the effect of *I*_h_ channels we made recordings from eight cells (four from Wistar rats, four from Sprague-Dawley; the data were indistinguishable and were pooled). We applied the same combination of square-pulse and naturalistic stimuli as outlined above in control and then after applying ZD7288 to block *I*_h_ channels (25 *μ*M; [34]). The effects of ZD7288 reached steady state after ∼ 10 min.

### Model extraction from experiment

The experimental dynamic *I-V* method (for a full description see reference [32]) generates neuron models of the non-linear integrate-and-fire kind with voltage *V* dynamics obeying

$$\begin{array}{c} \frac{dV}{dt}=F\left(V\right)+I_{\text{in}}/C \end{array}$$ (1)

where *F*(*V*) is the forcing term proportional to minus the voltage-averaged *I-V* curve (Fig 1E) divided by the cellular capacitance *C* and *I*_in_ is the injected fluctuating current. The method assumes that the voltage dynamics are of the form $C\dot{V}+I_{\text{ion}}=I_{\text{in}}$ so that the ionic current as a function of time can be found by subtracting the derivative of the measured voltage from the known injected current $I_{\text{ion}}(t)=I_{\text{in}}-C\dot{V}$ where the capacitance itself is extracted by a variance minimisation technique [32]. The average value of *I*_ion_ when the neuron is at a particular voltage *V*(*t*) defines the dynamic *I-V* curve *I*_dyn_(*V*). This is itself related to the forcing term via *F*(*V*) = −*I*_dyn_(*V*)/*C* (see Fig 1). Empirically we found that *F*(*V*) was accurately fit by the exponential integrate-and-fire [35] form

$$\begin{array}{c} F\left(V\right)=\frac{1}{\tau }(E-V+\Delta_{T}e^{\left(V-V_{T}\right)/\Delta_{T}}) \end{array}$$ (2)

for all classes of pyramidal cell, where *τ* = *C*/*g* is the membrane time constant and *g* is the input conductance of the cell. The other parameters are the resting potential *E*, the spike sharpness Δ_T_ and the spike-onset threshold *V*_T_. Certain parameters were seen to have transient modifications to their values following an action potential. These transients were well-captured by the refractory exponential integrate-and-fire (rEIF) model [32]. The rEIF model features additional post-spike dynamics for the EIF parameters *g*, *E*, and *V*_T_ (Fig 1H) so that, following a spike, these parameters evolve as

$$\begin{array}{ccc} g & = & g_{0}+g_{1}e^{-\left(t-t_{\text{ref}}\right)/\tau_{g}}, \end{array}$$ (3)

$$\begin{array}{ccc} E & = & E_{0}-E_{1}e^{-\left(t-t_{\text{ref}}\right)/\tau_{E1}}+E_{2}e^{-\left(t-t_{\text{ref}}\right)/\tau_{E2}}, \end{array}$$ (4)

$$\begin{array}{ccc} V_{T} & = & V_{T0}+V_{T1}e^{-\left(t-t_{\text{ref}}\right)/\tau_{T}}, \end{array}$$ (5)

where *t* is the time since the peak of the previous spike and (in this context) *g*_0_, *E*_0_ and *V*_T0_ are the late-time (or equivalently, steady-state) values. The jumps in the parameter values *g*_1_, *E*_1_, *E*_2_ and *V*_T1_ are measured from the end of the refractory time, which is *t*_ref_ = 4ms after the spike peak.

### Parameter extraction

A total of 21 parameters were extracted from traditional square-pulse currents and the dynamic *I-V* method. Three parameters were measured from step-current stimuli: the input resistance *R*_in_, calculated from the gradient of the current-voltage relationship around rest (using the mean voltage over the last 200 ms of each 1 s current step); its reciprocal the input conductance *G*_in_; and the sag percentage, defined by the difference between the minimum voltage during a hyperpolarising current injection and the steady voltage (the last 200 ms of a 1 s current step) expressed as a percentage of the steady voltage response compared to rest (Fig 1C). From the dynamic *I-V* protocol the parameters extracted were: the capacitance *C*, resting membrane potential *E*, the membrane time constant *τ*, the spike sharpness Δ_T_ and the spike-onset threshold *V*_T_ (Fig 1F) as well as their refractory properties (Fig 1G, 1H) where relevant. Three further parameters were extracted from the response to fluctuating currents: the average action-potential amplitude, duration, and maximal rate of rise (from isolated spikes with a preceding inter-spike interval greater than 200 ms and averaged to give a value for that cell; Fig 1I). Amplitude was defined as the distance between the spike initiation threshold for that spike, calculated using the peak second-derivative method [36], and the maximum voltage reached. Duration was calculated as the width at half-maximum. Maximal rate of rise was the maximum voltage first derivative between action-potential initiation-threshold and peak. Additional compound parameters considered were the potential difference to threshold *V*_T_ − *E* and the steady current required for spike initiation *I*_spike_ = (*V*_T_ − *E*)/*R*_in_, both of which are measures of neuronal excitability.

### Model simulation

The rEIF model was simulated using a forward Euler scheme with a 50 *μ*s time step, corresponding to the 20 kHz acquisition rate of the experimental recordings. Action potentials were registered following a rapid rise in membrane potential with the integration stopped when *V* ≥ 30 mV (this is typically less than 0.7ms before the experimental spike peak, for cases where model and experiment have a closely agreeing subthreshold voltage 2ms prior to spike). Since the action-potential downswing is not explicitly modelled, the voltage integration was stopped for a refractory period of 4 ms, before restarting at a reset that was equal to the average experimental value at 4ms after the spike peak (typically above the pre-spike threshold *V*_*T*0_). The variables demonstrating a strong post-spike dynamics (*g*, *E*, and *V*_T_) were increased to their post-spike jump values and subsequently followed fixed post-spike dynamics given by Eqs (3)–(5) from *t* = *t*_ref_ = 4ms post-spike and onwards.

### Performance measures

We used a number of performance metrics to asses the quality of the fit. To compare spike trains we used a previously used coincidence measure [32, 37, 38] given by

$$\begin{array}{c} \Gamma =\frac{N_{\text{coinc}}-\left\langle N_{\text{coinc}}\right\rangle }{1/2(N_{1}+N_{2})}\frac{1}{𝓝}, \end{array}$$ (6)

where *N*_1_ is the number of spikes in the reference spike train, *N*_2_ is the number of spikes in the spike train for comparison, *N*_coinc_ is the number of coincidence spike occurrences between the two spike trains with a precision Δ (in this case Δ = 5 ms), ⟨*N*_coinc_⟩ = 2*f*Δ*N*_1_ is the number of expected coincidences generated by a homogeneous Poisson process with firing rate *f*, the rate of the reference spike train, and 𝓝 = 1 − 2*f*Δ is a normalising factor so the Γ = 1 corresponds to an exactly coincident spike train. Since neurons have some intrinsic unreliability two identical current inputs will not produce identical spike trains. As such we computed two Γ values: Γ_rep_, comparing the target experimental spike train to a repeat recording obtained 10 minutes later with same driving current, and Γ_sim_, comparing the result of simulating the rEIF model with the same driving current to the target experimental recording. The ratio Γ_sim_/Γ_rep_ compares the simulated spike train to the intrinsic reliability of the cell. Cells were discarded as intrinsically unreliable if Γ_rep_ < 0.75, a criterion failed by 13% of all cells analysed.

### Statistical analysis

The dataset used for final analysis consisted of 136 cells and 18 parameters (Table 1, excluding *G*_in_, *t*_sag_, and *t*_0_). Unless otherwise stated, pairwise comparisons were made with a Mann-Whitney’s U test at the 5% significance level, and means are quoted ± the standard error of the mean. To control for the familywise error rate we applied the Bonferroni correction for multiple comparisons. Prior to principal component analysis (to remove any bias towards high numerical values of a given parameter) log-normally distributed parameters were transformed to log-space, so that they were normally distributed, after which the dataset was normalised using the z-score *Z* = (*μ*_*i*_ − *X*)/*σ*_*i*_, where *μ*_*i*_ and *σ*_*i*_ are the mean and standard deviation, respectively, of parameter *i* over the entire dataset. To examine correlations both Spearman’s rank correlation test and standard covariance tests were used. All data and statistical analyses was performed with custom-written MATLAB scripts utilising the Statistics and Global Optimisation toolboxes.

**Table 1:** Table of parameters measured in this study, separated into (from top to bottom) subthreshold, firing, action potential shape, and post-spike groups.

**Parameter**	**Description**
*C*	Membrane capacitance (pF)
*R*_in_	Input Resistance (MΩ)
*G*_in_	Input conductance (nS)
*τ*	Membrane time constant (ms)
*E*	Membrane equilibrium potential (mV)
*S*	Sag percentage from hyperpolarising current

*V*_T_ − *E*	Potential between threshold and rest (mV)
*V*_T_	Spike-onset threshold (mV)
*I*_spike_	Spike initiation current (pA)

Δ_T_	Spike sharpness (mV)
*A*_amp_	Action potential amplitude (mV)
*A*_dur_	Action potential duration (ms)
*A*_rise_	Action potential rate of rise (mV/ms)

*g*_1_	Post-spike jump in conductance (nS)
*τ*_*g*_	Conductance decay time constant (ms)
*V*_T1_	Post-spike jump in spike threshold (mV)
*τ*_T_	Spike threshold decay time constant (ms)
*E*_jump_	Post-Spike jump in *E* (mV)
*E*_sag_	Post-spike sag in *E* (mV)
*t*_sag_	Post-spike time of *E*_sag_ (ms)
*t*_0_	Post-spike time at which *E* crosses baseline (ms)

### Supporting Information and full data archive

Together with this paper we provide algorithms in the form of MATLAB scripts for generating parameter sets (for each of the pyramidal-cell populations considered) with the experimentally measured marginal distributions and crosscorrelations. The first script Generate_EIF.m provides parameter sets for the standard EIF model [35] for *C*, *τ*, *E*, *V*_T_ and Δ_T_ (S1 Computer Code). The second script Generate_rEIF.m also generates the additional quantities required for the full refractory EIF model [32] including the parameterization of the post-spike dynamics of *E*, *g* and *V*_T_ (S2 Computer Code). We also provide the MATLAB code DynamicIVAnalysis.m used for the dynamic IV method together with two pairs of data sets—one for extracting the parameters and one for testing the resulting model (S3 Computer Code and Data Files). All three scripts are published under the GNU General Public Licence, Version 3 (http://www.gnu.org/copyleft/gpl.html). Finally, the full data set comprising whole-cell patch-clamp recordings from 136 pyramidal cells stimulated by square-wave and fluctuating current protocols has been published at www.datadryad.com (doi:10.5061/dryad.d30k7).

## Results

Whole-cell patch-clamp recordings from 136 somatosensory-cortex pyramidal cells were made across layer 2/3 to layer 5 (Fig 1A). We initially classified the cells based on laminar location and somatic size under DIC infrared microscopy, and also examined the spiking pattern in response to an initial step-current protocol (Fig 1B-1C). Four distinct classes of pyramidal cell were identified and recorded from: layer 2/3 (L2/3, *n* = 31), layer 4 (L4, *n* = 29), slender-tufted layer 5 (SL5, *n* = 29) and thick-tufted layer 5 (TL5, *n* = 47) corresponding to previous classifications [1, 15, 17]. We then proceeded to extract electrophysiological parameters, analyse their distribution and examine the quality of the associated neuron models that were generated. The post-recording morphology of the cell, seen under confocal microscopy using fluorescent-dye filling or biocytin staining, was used as an additional criterion for identifying pyramidal-neuron class.

### Parameter measurement

Parameters were extracted from responses to square-pulse currents and fluctuating *in-vivo*-like stimuli (see Methods). A total of 21 parameters were used (Table 1) and divided into four groupings: subthreshold, firing, action-potential shape and post-spike parameters.

From step-current injections were measured input resistance (with its reciprocal the input conductance) and sag depth in response to a hyperpolarising current step (Fig 1C). Additional parameters, together with their post-spike dynamics, were extracted from the response to fluctuating stimuli using the dynamic *I-V* protocol (see Methods and reference [32]). During fluctuating current injection, the timecourse of the ionic current was found from the difference between the injected stimulation current and the capacitive current $I_{\text{ion}}(t)=I_{\text{in}}-C\dot{V}$ (Fig 1D) where the membrane capacitance *C* is found using a variance-minimisation technique [32]. The instantaneous current-voltage relationship, or dynamic *I-V* curve is then found from the average value of *I*_ion_ at a particular voltage *I*_dyn_(*V*) = ⟨*I*_ion_(*V*, *t*)⟩_*V*_ (Fig 1E). This projection of the time-dependent ionic current onto a quantity that varies only with the (time-dependent) voltage allows for direct generation of neuron models of the non-linear integrate-and-fire type $\dot{V}=F(V)+I_{\text{in}}/C$, with the forcing function *F*(*V*) related to the best estimate of the instantaneous ionic current through *F*(*V*) = −*I*_dyn_(*V*)/*C* (Fig 1F). Empirically we found that the forcing function *F*(*V*) was very well fit by the exponential integrate-and-fire form [35] given by Eq (2) for all classes of pyramidal cell in layers 2/3, 4 and 5. This form is parameterised by the membrane time constant *τ*, the resting potential *E*, the spike sharpness Δ_T_ and the spike-onset threshold *V*_T_. These four constants, together with the capacitance (providing a second estimate of the conductance, via *g* = *C*/*τ*, in close agreement with that derived from square-pulse protocols) characterise the response of the neuron for times that were greater than 200 ms after an action potential, i.e. after any spike-triggered transients have dissipated.

During a spike the transient activation and inactivation of transmembrane currents cause a rapid change and then relaxation back to baseline of the average ionic current. The timecourse can be measured using the dynamic *I-V* method in spike-triggered mode (Fig 1G) with the parameters of the changing forcing function *F*(*V*, *t*) typically exhibiting a jump followed by exponential or bi-exponential relaxation back to their steady-state values over tens of milliseconds (Fig 1H). The only parameters exhibiting significant transients were: the membrane conductance *g* (calculated from *C*/*τ*) which decayed mono-exponentially; the equilibrium potential *E*, which typically decayed bi-exponentially; and the spike-onset threshold *V*_T_, which decayed mono-exponentially (Fig 1H). There was little variability, with no consistent trend, seen in the spike sharpness parameter Δ_T_. It should also be noted that this quantity is an effective parameter that is larger than the true value of the spike sharpness for individual spikes. This is due to the dynamic *I-V* curve method averaging over many spikes with variations in the threshold that arise from membrane voltage fluctuations in the run up to the spike [39–41]. The post-spike dynamics of *g*, *E* and *V*_T_ were modelled by exponential functions yielding a number of additional parameters (see Methods Eqs (3)–(5)). The action-potential shapes, characterised by amplitude, duration and rise, were also extracted from the voltage-response to fluctuating current stimulation (Fig 1I).

As well as the directly measured parameters just described, hybrid parameters characterising excitability were also defined and comprised the potential difference between spike-onset threshold and rest *V*_T_ − *E* and the spike-initiation current *I*_spike_ = (*V*_T_ − *E*)/*R*_in_.

### Quality of model fits

**Fig 2:**
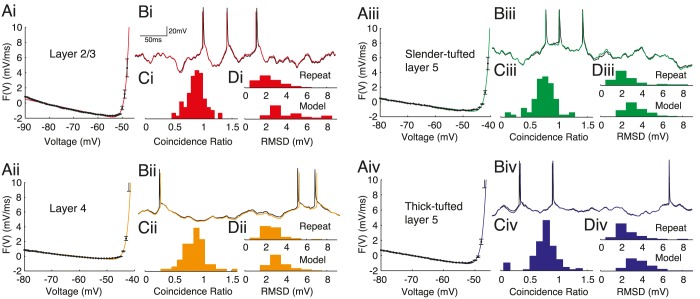
The dynamic *I* − *V* curve and associated refractory Exponential Integrate-and-Fire (rEIF) model fits to (i) layer-2/3, (ii) layer-4, (iii) slender-tufted layer-5 and (iv) thick-tufted layer-5 pyramidal-cell voltage timecourses. For each population the first panel **A** shows representitive empirical steady-state forcing terms (black; proportional to minus the dynamic *I-V* curve divided by capacitance) with the EIF form (colour; Eq (2)) providing a good fit for all populations. **B** compares the accuracy of the resulting rEIF model (coloured) to an experimental voltage trace (black). The histograms show performance measures for the voltage-timecourse fits: the spike-coincidence ratio **C** and the root-mean-squared difference in voltage **D** between repeated experimental traces (upper histogram), and experiment and model traces (lower histogram).

We found that the empirical dynamic *I-V* curve could be accurately fitted by the exponential integrate-fire (EIF) form Eq (2) for each of the four pyramidal-cell classes from layers 2/3, 4 and 5 (Fig 2Ai-2Aiv). To quantify the quality of the extracted model we compared (Fig 2Bi-2Biv) the neuronal response to novel stimuli with the prediction from a simulation of the refractory EIF (rEIF) model, an extension [32] of the standard EIF model [35] to include post-spike parameter dynamics (Eqs (3)–(5)). The rEIF model gave a good account of the spike times with mean(std) coincidence ratios, which compare the similarity of the model and experimental spike trains to the intrinsic cell reliability (see Methods), of L2/3 0.88(0.16), L4 0.86(0.20), SL5 0.74(0.21) and TL5 0.76(0.22), with an overal value of 0.81(0.21) for all cells (Fig 2Ci-2Civ). We also compared the subthreshold response of the model and experiment (Fig 2Di-2Div) with the root mean squared-deviation (RMSD) of the set of data points excluding 2 ms before and 4 ms after a spike peak. The RMSDs between model and experiment were L2/3 4.6(2.8)mV, L4 3.4(1.1)mV, SL5 3.4(1.0)mV and TL5 3.8(1.2)mV with an overal value of 3.8(1.7)mV. These values can be compared to the RMSD for experiments where the stimulus was repeated: L2/3 2.5(1.1)mV, L4 2.9(1.6)mV, SL5 2.7(1.6)mV and TL5 3.0(2.0)mV, with an overall value of 2.8(1.7)mV (an effective accuracy of ∼ 1 mV between the rEIF model and experiment).

### Post-spike parameter response

**Fig 3:**
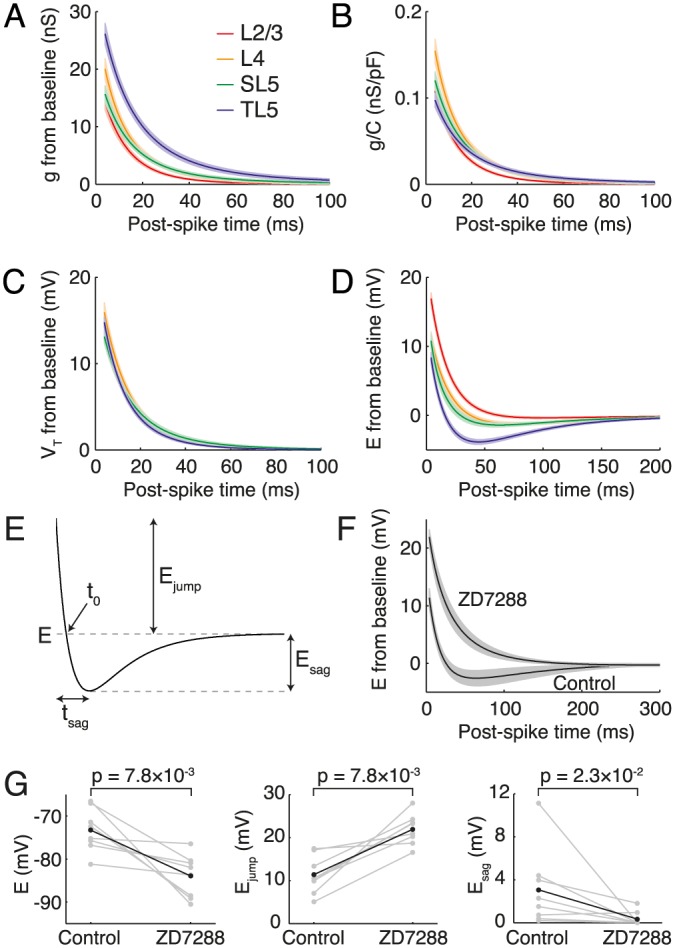
Class-dependent post-spike dynamics of **A** conductance *g*, **B** *g*/*C* = 1/*τ*, **C** spike threshold *V*_T_ and **D** equilibrium potential *E*. Note the sag in the *E* dynamics of TL5 cells. **E** Quantifying *E* dynamics by jump and sag depth. **F** TL5 *E* dynamics in control conditions and after application of *I*_*h*_-blocker ZD7288, which abolished the sag. **G** The blocker also resulted in a hyperpolarization of the steady-state rest, with a post-spike jump increase and sag-depth reduction.

Before going on to analyse the parameter distributions across the pyramidal-cell populations, we examined the post-spike parameter dynamics in more detail. Fig 3 shows the mean timecourses of conductance *g*, resting potential *E* and spike-onset threshold *V*_T_ for each of the four cell classes. Considering first the post-spike conductance dynamics, thick-tufted layer-5 (TL5) pyramidals had a greater conductance increase (Fig 3A) immediatelty following a spike. However, on normalization by the capacitance (Fig 3B) the disparity is weaker indicating that the higher jump in conductance in TL5 pyramidal cells is due to size rather than to differences in channel properties or density. The post-spike dynamics of the spike-onset threshold *V*_T_ showed little difference between the four classes (Fig 3C): all showed a significant jump, between 10–20 mV, in the threshold for action-potential generation. The post-spike dynamics of the instantaneous resting potential *E* were markedly different between pyramidal-cell populations: L2/3 cells displayed a mono-exponential response whereas TL5 pyramidals featured a significant sag, with the other two classes intermediate (Fig 3D). To conveniently characterise the dynamics of *E* we introduce four new parameters: the post-spike jump in the equilibrium potential *E*_jump_, the depth of the subsequent sag *E*_sag_, the time to sag *t*_sag_ and the interception time *t*_0_ (Fig 3E). It was previously proposed that the sag in the equilibrium potential *E*(*t*) post-spike dynamics [32] is the result of a transient inactivation of the h-current during the spike followed by its subsequent re-activation at more hyperpolarized voltages [42]. To test this hypothesis we measured the post-spike dynamics of *E* in TL5 cells in control conditions and following the application of 25 *μ*M of the *I*_*h*_-channel blocker ZD7288 [34]. On blocking *I*_*h*_ channels the bi-exponetial sag dynamics changed to a mono-exponential response (Fig 3F). The drug application also resulted in significant membrane hyperpolarisation, an increase in the relative post-spike resting-potential jump and a reduction or complete abolition of the post-spike sag (Fig 3G) supporting the hypothesis that *I*_*h*_ channels underlie the non-monotonic post-spike timecourse seen in TL5 pyramidal cells.

### Differences between cell classes

**Fig 4:**
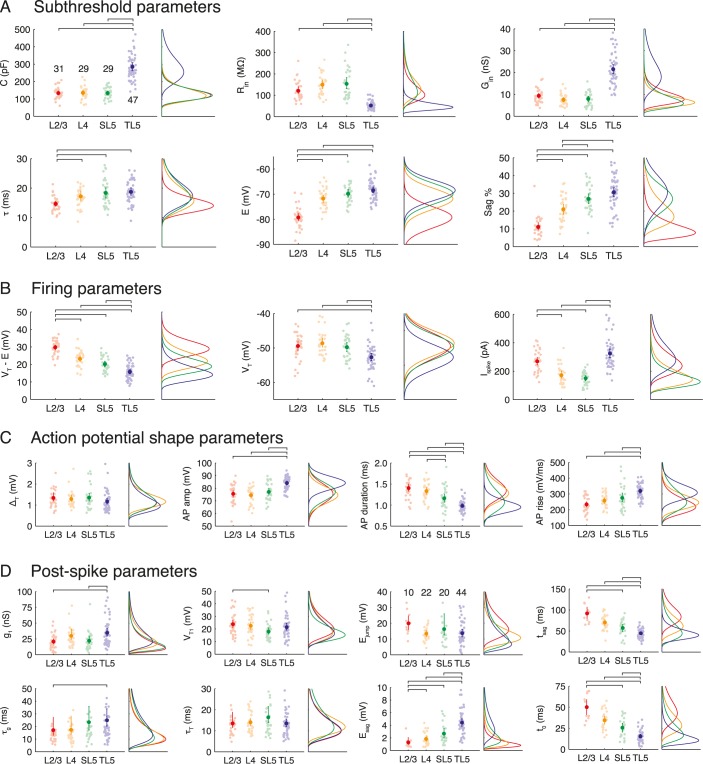
Quantifying heterogeneity in neocortical pyramidal-cell populations. The parameter groupings are: **A** subthreshold, **B** firing, **C** action-potential shape and **D** post-spike parameters for each pyramidal-cell class. Left panels show mean value (darker point) and all data (pale points) with error bars (95% bootstrap confidence interval). Horizontal lines denote significant statistical difference (5% confidence, Bonferroni-corrected). Right panels show fitted distributions for each pyramidal-cell class. The *n* numbers are shown on the first panel and are common to all other panels except the four concerning the resting-potential sag dynamics *E* for which only those cells with sags were included (*n* numbers are given in the *E*_jump_ panel). Note that many parameters correlate with cortical depth.

All data for the four pyramidal-cell classes studied for each of the 21 parameters (Table 1) are shown in Fig 4 together with the fitted distributions (see also Table 2). Pairwise significance tests were performed between cell classes for each parameter to identify significant differences and are shown when above the 5% level. We now consider the major features of the data set by parameter groupings.

**Table 2:** The mean *μ* and standard deviation *σ* for the parameter distributions (log-normal unless otherwise marked).

**Parameter**	**Layer 2/3**		**Layer 4**		**Slender Layer 5**		**Thick Layer 5**	
	*μ*	*σ*	*μ*	*σ*	*μ*	*σ*	*μ*	*σ*
*C* (pF)	134	32.8	135	36.7	133	31.9	284	78.5
*R*_in_ (MΩ)	121	45.2	149	50.5	154	73.3	52.4	19.4
*G*_in_ (nS)	9.31	3.24	7.52	2.69	7.94	3.38	21.5	7.40
*τ* (ms)	14.6	2.53	17.2	4.18	18.3	4.74	18.7	4.23
*E* (mV)	−79.3*	4.27*	−71.8*	4.20*	−69.9*	4.18*	−68.5*	3.98*
*S*(%)	11.0	5.70	21.0	7.65	26.8*	7.58*	30.6*	9.20*

*V*_T_ − *E* (mV)	29.8	4.16	23.2	4.46	20.1	4.28	15.7	4.25
*V*_T_ (mV)	−49.5*	3.81*	−48.7*	3.53*	−49.7*	3.56*	−52.7*	3.59*
*I*_spike_ (pA)	270	77.4	173	66.5	150	48.6	324	105

Δ_T_ (mV)	1.34	0.550	1.28	0.394	1.35	0.523	1.16	0.479
*A*_amp_ (mV)	75.5*	7.76*	74.5*	6.47*	77.2*	7.07*	84.1*	5.15*
*A*_dur_ (ms)	1.41	0.245	1.33	0.215	1.16	0.271	0.981	0.149
*A*_rise_ (mV/ms)	234	50.3	258	46.2	275	76.8	319	46.9

*g*_1_ (nS)	14.3	7.5	20.0	9.6	15.7	7.7	26.1	12.5
*τ*_*g*_ (ms)	17.0	17.4	17.3	15.8	23.3	23.9	24.5	21.8
*V*_T1_ (mV)	16.2	4.4	15.9	5.1	13.1	4.0	14.8	4.4
*τ*_T_ (ms)	13.6	8.9	14.1	4.9	16.4	9.7	12.7	5.3
*E*_jump_ (mV)	15.8	5.7	10.1	3.7	9.6	4.3	7.9	4.6
*E*_sag_ (mV)	1.3	0.9	1.8	1.1	2.7	1.7	4.4	2.0
*t*_sag_ (ms)	87.5	23.2	66.2	18.9	53.0	19.9	40.4	11.5
*t*_0_(ms)	45.9	15.7	30.6	12.2	21.6	12.0	11.5	6.8

* Normal distribution.

The main observations for the subthreshold parameters (Fig 4A) were: L2/3 cells are well distinguished by their short time constant, hyperpolarised equilibrium potential and lack of sag response to a hyperpolarising step current; TL5 pyramidal cells are distinguished by large capacitance and conductance, as expected, and further separated from the layers 2/3 and 4 populations by their depolarised resting potential and strong sag response; the only significant difference between L4 and SL5 cells is their sag percentage, with SL5 cells displaying a statistically significant larger response (one of only two parameters to distinguish between these cell classes).

For the firing parameters (Fig 4B) it was seen that: the potential difference between rest *E* and spike-onset threshold *V*_*T*_ decreases monotonically from 30mV to 16mV from L2/3 to L5 with the majority of the change due to an increasingly depolarised rest rather than significant change in spike-onset threshold *V*_T_; TL5 spike-onset threshold is significantly lower than in other layers; on normalising the potential difference to threshold by the membrane resistance, L2/3 and TL5 cells are both well distinguished from L4 and SL5 cells by their requirement of a higher current for spike initiation.

For the action-potential shape parameters (Fig 4C): no difference was seen across pyramidal-cell populations in the spike sharpness parameter Δ_T_; however, TL5 cells separate well in their higher amplitude (consistent with lower *V*_*T*_), shorter duration, and greater maximal rate of rise; spike duration also discriminates between L4 and SL5 cells (the second of the two parameters in our dataset to do so).

For the post-spike parameters (Fig 4D): TL5 pyramidals have a greater spike-triggered jump in conductance than L2/3 and SL5 cells (due to their greater size: see Fig 3B and related discussion in the main text) and a significantly longer *τ*_*g*_ than L2/3 cells; the only significant difference in spike-onset threshold was the greater jump of L2/3 compared to SL5 cells; however, the resting-potential dynamics displayed a more marked difference between classes as already discussed (Fig 3D). Considering first only the cells from each class that display a sag in the post-spike response (defined as *E*_sag_ > 0.5mV; *n*-numbers given in the Fig 4C panel for *E*_jump_), L2/3 cells are well separated by their smaller sag depth whereas TL5 cells have a significantly greater sag depth than all other classes. These observations are consistent with the sag percentages during hyperpolarising square-pulse current stimuli for the different pyramidal-cell classes (Figs 1C and 4A) given that *I*_*h*_ underlies this response (Fig 3F). Each of the four cell classes had a different proportion that displayed a post-spike sag in resting potential (L2/3 0.32; L4 0.76; SL5 0.69; TL5 0.94). Cells (*n* = 40) that did not exhibit the sag response could all be described by a mono-exponential drawn from a single class-independent distribution with a post-spike jump *E*_mono_ and decay-time constant *τ*_mono_ (see next paragraph).

We fitted distributions to each parameter for the four pyramidal-cell classes to enable the generation of reduced neuron-models with the correct marginal distributions (parameter covariance is considered later). All parameters were well fitted by log-normal distributions except: the resting potential *E*, spike-onset threshold *V*_T_, action-potential amplitude *A*_amp_ and sag percentage *S* for both classes of layer-5 cell, which were instead fitted to normal distributions. Parameters of the fitted distributions are summarised in Table 2 with the exception of the log-normal distributions for *E*_mono_ and *τ*_mono_ that have mean and standard deviations (*μ*, *σ*) of (16.1, 4.8) mV and (15.1, 4.5) ms, respectively.

### Variability between and within cell classes

**Fig 5:**
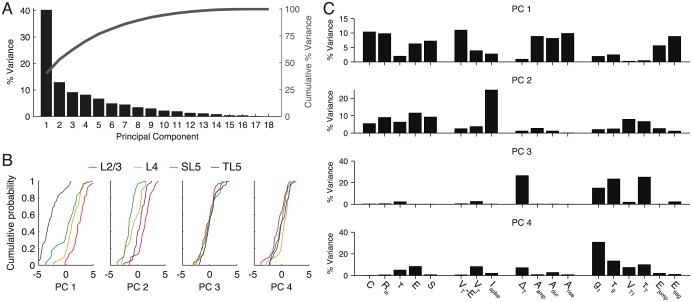
Principal component analysis (PCA) of variation across the entire data set. **A** Pareto plot of the percentage of the variance explained by each principal component (black bars), and the cumulative sum of the explained variance (dark grey line). **B** Empirical cumulative distribution function in PC space for each class and for principal components one to four. **C** Contribution of each cellular parameter to the variation of the first four principal components. See main text for an interpretation of these results.

To determine the major sources of variability in the full dataset we used principal component analysis (PCA). As seen in Fig 5A, the first principal component (PC) explained 40% of the variance; however, no small additional number of PCs explained the remainder, with 9 components required to explain 90% of the variance.

Fig 5B shows the empirical cumulative distribution functions of the first four PCs with the percentage of variance explained by each variable within each of the first four PCs provided in Fig 5C. From the cumulative distributions (Fig 5B) it can be seen that the first PC strongly discriminates between TL5 pyramidal cells and the remaining classes, with some discriminatory power for L2/3 cells. Fig 5C shows that PC1 is primarily determined by the steady-state properties of the cell: the subthreshold properties, distance to threshold and action-potential shape, though there is also a contribution from the the jump and depth of the post-spike sag in the resting potential arising from the h-current. The second PC weakly discriminates between pyramidal-cell classes (Fig 5B) and is mainly determined by an excitability measure: the spike-initiation current, which accounts for more than 20% of the variance within this PC. The third PC is determined by the spike sharpness parameter Δ_T_ and, as for the fourth PC, the post-spike dynamics of conductance *g* and spike-onset threshold *V*_T_. Neither PC3 or PC4 discriminates well between cell classes and so the statistics of the spike-sharpness parameter Δ_T_, and post-spike dynamics of *g* and *V*_T_ are common to all classes of pyramidal cell.

**Fig 6:**
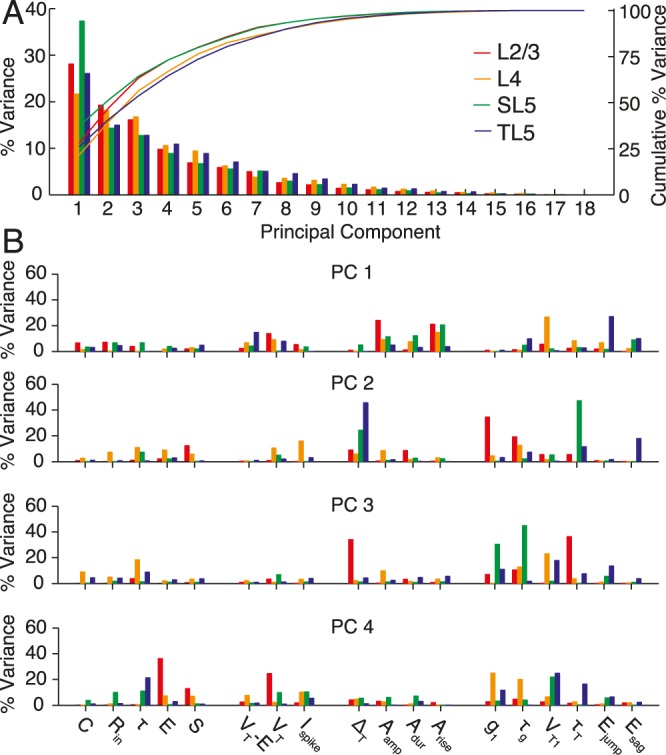
Principal-component analysis (PCA) for the data from each pyramidal-cell population. **A** Pareto plot of the percentage of variance explained by each principal component when PCA was performed on each class individually. **B** Contribution of each parameter to the variation of the first four principal components for each pyramidal-cell population.

To investigate the sources of variability within a cell class we performed PCA on each class separately. The percentage of variance explained by each PC for each cell class is shown in Fig 6A, and the within-PC variation explained by each parameter is shown in Fig 6B. The PCs were significantly different for the four classes of cells with the principal sources of variability being: for L2/3 cells the action-potential amplitude and rise, and post-spike conductance parameters; for L4 cells *A*_rise_ and *V*_T1_; for SL5 cells the action-potential shape parameters; and for TL5 cells the post-spike dynamics of the resting potential and potential difference to threshold.

### Correlations between neuronal parameters

**Fig 7:**
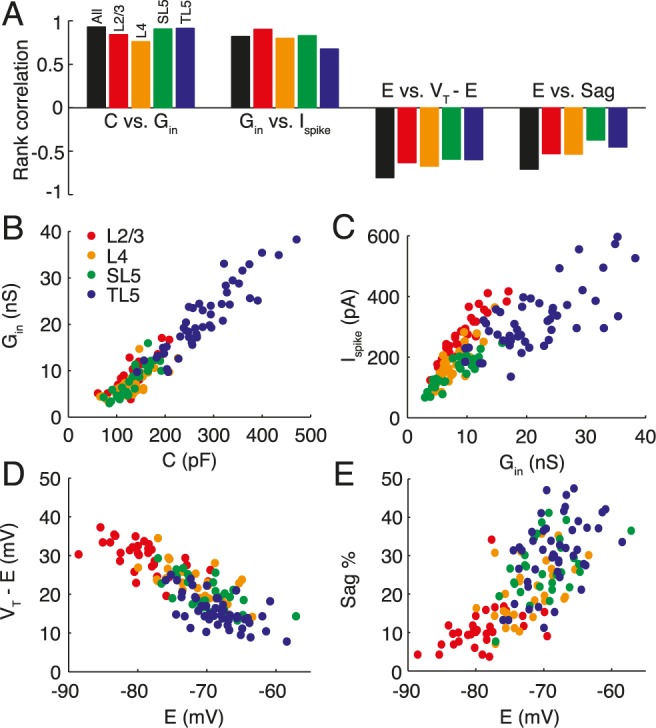
Correlations between pairs of electrophysiological parameters. **A** Highly correlated variable pairs for each pyramidal-cell population (see colour key) and across all pyramidal cells (black). Correlations were calculated using Spearman’s rank test. As well as the correlations directly linked to size such as **B** conductance versus capacitance or, less directly, **C** the spike-threshold current versus conductance, there are **D** anti-correlations between the potential difference to threshold and the resting potential and **E** positive correlations between the sag percentage and resting potential, the latter of which is likely due to a common underlying cause such as *h*-current expression.

To search for correlations or anti-correlations in our data we performed Spearman’s rank correlation tests on all parameter pairs. Four parameter pairs with high Spearman’s rank correlation scores are shown in Fig 7A. The positive correlation between conductance and capacitance (Fig 7B) is due to the size of the cell’s surface area and more-or-less proportional expression of conductances. The positive correlation between the spike-threshold current *I*_spike_ = *G*_in_(*V*_T_ − *E*) and conductance (Fig 7C) demonstrates little compensation for cell size by decreased potential difference from rest to threshold (*V*_T_ − *E*), except for the thick-tufted layer-5 pyramidal cells that show a relatively lower required *I*_spike_ at higher conductances. The negative correlation between the potential difference to threshold *V*_T_ − *E* (Fig 7D) and resting potential *E* is a consequence of the weak variability in the absolute spike threshold *V*_T_ (Fig 4E), and the correlation between *E* and the sag percentage (Fig 7E) is consistent with the dual role of *I*_*h*_ in generating a more depolarised resting potential and the strong sag dynamics from delayed negative feedback.

Given the log-normality or normality of the parameters we tested whether the empirical parameter distributions (with logarithms taken of the log-normally distributed variables) were consistent with having been drawn from a multivariate Gaussian distribution using two tests (Henze-Zirkler’s and Royston’s tests). The EIF parameter sets for all layers all passed at least one test at the 5% significance level with the only parameter sets to fail both tests at 5% being the full rEIF parameter set for layer-2/3 pyramidal cells and the mono-exponential parameter sets for the post-spike response of the equilibrium potential *E*(*t*). For reference the covariance matricies for the EIF parameters are provided in Table 3 together with the Spearman’s rank correlation values. The normalised covariance matricies for the full parameter sets can be found in the script Generate_rEIF.m provided in the Supporting Information.

**Table 3:** Covariance (upper triangle and diagonal) and Spearman’s rank correlation (lower triangle only as diagonals would be unity) of the appropriately transformed Exponential Integrate-and-Fire model parameters log(*C*/pF), log(*τ*/ms), *E* (mV), *V*_T_ (mV) and log(Δ_T_/mV) for each of the four cell classes. The transformed parameters have normal marginal distributions and the combined sets satisfy at least one test for multivariate normality at the 5% level (see main text). All values are given to two significant figures.

	**Layer 2/3**					**Layer 4**				
	log(*C*)	log(*τ*)	*E*	*V*_T_	log(Δ_T_)	log(*C*)	log(*τ*)	*E*	*V*_T_	log(Δ_T_)
log(*C*)	0.066	−0.012	0.0047	−0.55	−0.012	0.083	0.0078	0.25	−0.25	−0.010
log(*τ*)	−0.41	0.029	−0.099	0.28	−0.028	0.19	0.058	0.28	−0.0045	−0.0066
*E*	0.11	−0.22	18	7.8	−0.071	0.20	0.067	18	5.1	−0.38
*V*_T_	−0.48	0.35	0.38	15	−0.42	−0.14	−0.025	0.29	12	−0.38
log(Δ_T_)	0.13	−0.43	−0.0016	−0.39	0.13	−0.13	−0.014	−0.30	−0.47	0.071
**Slender-tufted layer 5**						**Thick-tufted layer 5**				
log(*C*)	0.063	0.022	0.49	−0.15	−0.02	0.075	0.004	0.16	−0.28	−0.021
log(*τ*)	0.34	0.065	0.28	−0.031	−0.019	0.031	0.052	0.070	−0.0054	−0.017
*E*	0.49	0.14	17	5.9	−0.65	0.16	0.066	16	5.3	0.22
*V*_T_	−0.22	−0.086	0.35	13	−0.18	−0.33	0.088	0.29	13	0.25
log(Δ_T_)	−0.20	−0.21	−0.23	−0.039	0.13	−0.20	−0.070	0.18	0.27	0.13

### Heterogeneous populations of model neurons

**Fig 8:**
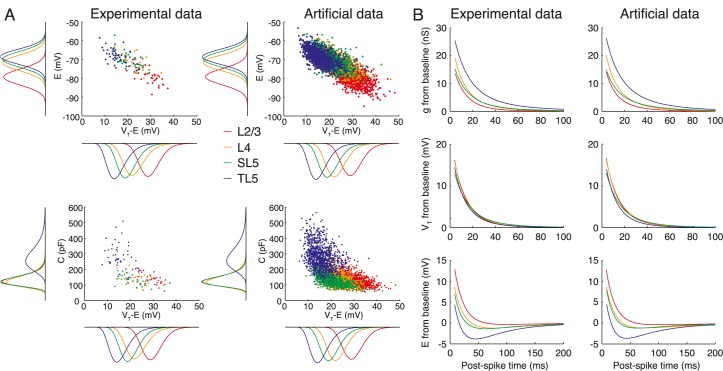
Experimental data compared to the algorithm that generates parameter sets for heterogeneous populations of L2/3-L5 model neurons with correct marginal distributions and correlation structure. **A** Relationship between steady-state resting potential *E* and potential difference to threshold *V*_T_ − *E* (top), and capacitance *C* and potential difference to threshold *V*_T_ − *E* (bottom) for experimental (left) and algorithmically generated (right) datasets. **B** Corresponding mean response for the post-spike dynamics of *g*, *V*_T_, and *E* in the experimental and algorithmically generated datasets. The artificial dataset used here comprised 1000 cells for each of the four classes.

A central aim of this study was to provide algorithms to generate sets of neuron models with experimentally verified marginal distributions and covariance structure in their parameters. Together with this publication we provide two algorithms. The first generates parameter sets for the standard EIF model [35] comprising parameters *C*, *E*, *τ*, *V*_T_ and Δ_T_. The method uses a multivariate Gaussian sampler (implemented using a Gaussian copula) for the combination of logarithmed log-normally-distributed variables and normally distributed variables: the normalised covariance matricies for these quantities can be found in the MATLAB script Generate_EIF.m provided in the Supporting Information (S1 Computer Code: the covariance between the non-logarithmed variables can be found in Table 3). A second MATLAB script Generate_rEIF.m also provided in the Supporting Information (S2 Computer Code) generates parameter sets for refractory EIF models that additionally feature the empirical post-spike dynamics seen in parameters *g*, *V*_*T*_ and *E*. In Fig 8 we demonstrate our algorithms by generating 1000 parameter sets for each class of pyramidal cell. The correlation structure in the simulated data set and the marginal distributions are adhered to, for example in *E* vs. *V*_T_ − *E* space (Fig 8A top) and *C* vs. *V*_T_ − *E* space (Fig 8A bottom). Furthermore, on implementation of the associated rEIF model Eqs (3)–(5) the mean refractory response of *g*, *V*_T_, and *E* are indistinguishable from the mean responses of the cells in our data set (Fig 8B).

## Discussion

From the response to square-pulse and fluctuating current stimuli, we quantified the distributions and correlations in the passive, active and spike-triggered perisomatic electrophysiological parameters in populations of pyramidal cells in layer 2/3, 4 and the slender-tufted and thick-tufted cells of layer-5 of the juvenile rat somatosensory cortex. By measuring the dynamic *I-V* curve we found that a forcing term *F*(*V*) of the exponential integrate-and-fire kind [35] provided an accurate fit to the somatic current-voltage relation for all classes of pyramidal cell from layers 2/3-5, extending the applicability of the EIF model from earlier studies on thick-tufted layer-5 pyramidal cells [32], fast-spiking GABAergic interneurons [43] and striatal medium spiny neurons [44].

Significant differences were seen in the average parameter values between pyramidal-cell populations as well as significant spreads of parameter values within each population. On average L2/3 cells had a shorter membrane time constant and more hyperpolarised membrane potential (by 10mV) than TL5 neurons, with the sag response to step-current input or an action potential (mediated by *I*_*h*_) increasing from layers 2/3 to 5. As regards the excitability measures, the typical potential difference to threshold from rest *V*_T_ − *E* decreased by ∼ 15mV from L2/3 cells to TL5 cells; however, on normalization by the input resistance to give the steady current *I*_*spike*_ required to bring the neuron to threshold, L2/3 and TL5 cells were seen to be similarly (despite their very different size) less excitable than L4 and SL5 cells.

Principal component (PC) analyses demonstrated that, apart from the first component (determined by subthreshold properties, distance to threshold, action-potential shape and the h-current mediated post-spike sag response) the subsequent components did not show a clear cut-off in their contribution. The first PC provided a good discrimination for L2/3 and TL5 from the other two populations (pairwise significance testing also showed clear differences between L2/3 and TL5 cells); however, L4 and SL5 pyramidal cells were almost indistinguishable in their electrophysiology, despite being located at different cortical depths, forming distinct morphological sub-groups [45, 46], and receiving input from different regions [47]. Only two of the 21 parameters, sag percentage and action potential duration (Fig 4), showed a significant difference for L4 and SL5 above the 5% threshold. We also provided an analysis for parameter covariability with the main non-trivial correlations arising from effects common to the *h* current such as depolarised threshold potential and the existence of a sag/rebound response.

### Extensions

The majority of parameters were measured using the dynamic-*I-V* method [32] that extracts a clearly defined biophysical quantity (the voltage-averaged instantaneous current-voltage relationship) and uses it to construct a non-linear integrate-and-fire model that accurately captures the integration of current flowing into the soma. An attractive feature of this class of reduced neuron model is that they are amenable to mathematical network analysis [12, 48], which is a powerful tool for studying the general properties of neuronal networks. Empirically, the forcing term of the exponential integrate-and-fire model [35] was found to provide a good fit to the steady-state *I-V* curves with the post-spike *I-V* dynamics captured by an extension to the EIF of the refractory EIF form [32]. Given the mathematical simplicity of the rEIF (it is a renewal process in the sense that post-spike parameter changes are non-cumulative) it is surprising that it provides such an accurate fit to experimental data (see Fig 2). A great variety of simplified neuron models have been proposed that could also have been used as generators of voltage timecourses in this study. Our preference for the dynamic-*I-V* approach to model construction is that few assumptions for the structure of the model need be made, because the components are directly extracted from experiment. This approach is distinct from those where a neuronal model (whether biophysically grounded or heuristic) is chosen *a-priori* and then the parameters are optimised by comparing model and experimental voltage timecourses. Alternative frameworks or extensions of the rEIF model that include additional state variables to better capture the effects of voltage-gated or calcium-gated currents, cumulative spike-frequency adaptation or cumulative spike-triggered parameter changes, spike-threshold variability or morphology (using multiple sites of dendritic stimulation), should lead to models with more predictive power. However, any model that aims to accurately capture the spiking dynamics of pyramidal cells does need to account for the high post-spike threshold and reset: this is not a feature of models that have the standard integrate-and-fire spiking mechanism nor is it a feature of more detailed models that use the standard Hodgkin-Huxley spike-generating currents. To facilitate further investigation along these lines by other research groups we have provided our full data set with this publication (www.datadryad.com; doi:10.5061/dryad.d30k7). Finally, in this study we restricted the measurements and analysis to pyramidal populations from layers 2/3, 4 and 5. An obvious extension of the current study would be to examine the heterogeneity in the diverse populations of layer 6 of the neocortex. The layer comprises three major pyramidal-cell classes [49] that can be divided into further morphological subgroups. Quantifying the electrophysiological properties of the different populations, using a broad-band stimulus that mimics aspects of *in-vivo* synaptic drive like the dynamic *I-V* method, would allow for the development of a useful tool for rapid on-line classification of layer-6 pyramidal cells during intracellular recording.

### Network models with heterogeneity

The primary outcome of this work was to provide a tool to aid the analyses of the significant levels of heterogeneity seen in neocortical pyramidal-cell populations. Our algorithms for generating artificial datasets adhere to the experimentally determined marginal distributions and correlation structure of parameter space (Fig 8). Coupling this work to other studies that quantify diversity in the synaptic dynamics, connectivity and topology will allow network models to be constructed in which the heterogeneities of neocortical cell populations are respected. Network analyses are beyond the scope of this paper, but it has already been demonstrated that various forms of heterogeneity can significantly affect synchrony [28], coding [30] and the neuronal gain [27] in a network context. Using the empirical statistics for parameter variability and correlation presented here will allow for a quantitative analyses of the effects of heterogeneity using reduced-models of neocortical pyramidal cells. It is hoped that such modeling will contribute to our understanding of how heterogeneity in network architecture affects processing of information in neocortical microcircuits and neuronal tissue more generally.

## Supporting Information

S1 Computer Code The MATLAB script Generate_EIF.m generates parameter sets for the basic exponential integrate and fire model [35] that respect the empirical marginal distributions and covariance. (M)

S2 Computer Code The MATLAB script Generate_rEIF.m generates parameter sets for the refractory exponential integrate and fire model [32] that respect the empirical marginal distributions and covariance. (M)

S3 Computer Code and Data Files The MATLAB script DynamicIVAnalysis.m uses the dynamic *I-V* curve method [32] to extract electrophysiological parameters from four example data files (current stimulations and voltage measurements) with the file prefix ExDynamicIVdata. (ZIP)
